# Multiancestry sex-stratified genomic associations with HIV viral load and controller status from the ICGH

**DOI:** 10.1172/jci.insight.170068

**Published:** 2023-06-08

**Authors:** Candelaria Vergara, Jeffrey F. Tuff, International Collaboration for the Genomics of HIV (ICGH), Jacques Fellay, Priya Duggal, Eileen P. Scully, Paul J. McLaren

**Affiliations:** 1 Johns Hopkins Bloomberg School of Public Health, Baltimore, Maryland, USA.; 2 National Laboratory for HIV Genetics, National Microbiology Laboratories, Public Health Agency of Canada, Winnipeg, Manitoba, Canada.; 3 See Supplemental Acknowledgments for ICGH details.; 4 Global Health Institute, School of Life Sciences, École Polytechnique Fédérale de Lausanne, Lausanne, Switzerland.; 5 Precision Medicine Unit, Biomedical Data Science Center, Lausanne University Hospital (CHUV) and University of Lausanne, Lausanne, Switzerland.; 6 Johns Hopkins University, Department of Medicine, Baltimore, Maryland, USA.; 7 Department of Medical Microbiology and Infectious Diseases, University of Manitoba, Winnipeg, Manitoba, Canada.

**Keywords:** AIDS/HIV, Genetics, Adaptive immunity, Complex traits, Genetic variation

## Abstract

Biological sex and host genetics influence HIV pathogenesis. Females have a higher likelihood of spontaneous viral control and lower set point viral load (spVL). No prior studies have assessed sex-specific genetics of HIV. To address this, we performed a sex-stratified genome-wide association study using data from the ICGH. Although it is the largest collection of genomic data in HIV, this multiethnic sample of 9,705 people is 81.3% male. We sought to identify sex-specific genetic variants and genes associated with HIV spVL and control. We confirmed associations in the *HLA* and *CCR5* regions in males and *HLA* in females. Gene-based analyses detected associations between HIV spVL and *PET100*, *PCP2*, *XAB2*, and *STXBP2* only in males. We detected variants with a significant sex-differential effect on spVL in *SDC3* and *PUM1* (rs10914268) and *PSORS1C2* (rs1265159) and on HIV control in *SUB1* (rs687659), *AL158151.3*, *PTPA,* and *IER5L* (rs4387067). Those variants have epigenetic and genetic interactions with relevant genes with both *cis* and *trans* effects. In summary, we identified sex-shared associations at the single-variant level, sex-specific associations at the gene-based level, and genetic variants with significant differential effects between the sexes.

## Introduction

Biological sex impacts HIV disease pathogenesis. Females generally have a ~0.35 log–lower viral load (1), lower levels of per-cell HIV RNA production by CD4^+^ T cells from lymph nodes (2), and lower levels of residual viral activity in the setting of viral suppression with antiretroviral therapy (ART) (3, 4). In addition, females are more likely to exhibit spontaneous control of HIV in the absence of ART (5–7). The mechanisms of differences between males and females are incompletely defined, with some studies suggesting a direct role for the sex hormone estrogen (8, 9). However, there has been limited exploration of the contribution of genetic factors to sex-based variation in disease progression.

The immune system has a sex-specific genetic architecture with gene dosage effects from sex chromosome–encoded immunoactive genes (10) and sex-specific regulation of autosomal genes across multiple immune cell subsets (11). Within this context, genetic variants can have 1 of the following effects in the 2 sexes: both sexes may show a similar effect, only 1 sex may show an effect, or both sexes may demonstrate an effect of the variant, but with substantial differences in the magnitude or direction of effect. In prior genome-wide association studies (GWASs) of infectious diseases, sex-differential autosomal variants and X chromosome associations have been described for a range of conditions, including COVID-19, smallpox, influenza, and tuberculosis (12–14). Moreover, hepatitis C is associated with sex-specific pathogenesis phenotypes, including an approximately 2-fold higher rate of spontaneous clearance in females, and a sex-stratified GWAS identified a male-specific autosomal region associated with hepatitis C clearance (15). In addition to autosomal genes, immunoactive genes on the X chromosome have the potential to contribute to infectious disease outcomes, as demonstrated by the association of *TLR7* gene variants with severe outcomes in SARS-CoV-2 infection in males (16).

Prior autosomal analyses have identified host genetic loci associated with both spontaneous HIV control and HIV set point viral load (spVL), but these analyses have not considered sex independently or included the sex chromosomes (17–20). The ICGH is the largest collection of genome-wide genetic data regarding people living with HIV (PLWH), with information including clinical outcomes on > 9,000 PLWH. The aim of this study was to assess whether genetic loci have sex-differential impacts on HIV spVL or spontaneous HIV control. We conducted a sex-stratified GWAS of autosomes and the X chromosome to identify sex-specific genetic variants associated with HIV spVL and HIV control phenotypes in PLWH of European and African descent participating in the ICGH study (19). We performed marker and gene-based analyses stratified by sex, tested the heterogeneity of the effect of top-associated variants between sexes, and compared the genetic effects of markers between the sexes.

## Results

### Cohort characteristics

To assess whether autosomal genetic variation impacts spVL in a sex-dependent manner, we accessed genotype and clinical data from 9,705 participants from the ICGH (19). This collaboration consists of a collection of HIV natural history of disease cohorts from 17 independent studies. The sample is predominantly composed of male participants (*n* = 7,890) and of participants of European ancestry (*n* = 6,315) with additional participants of African American (*n* = 2,535) and southern African (*n* = 855) ancestry (Supplemental Table 1; supplemental material available online with this article; https://doi.org/10.1172/jci.insight.170068DS1). Despite accounting for only 8% of the cohort, a higher proportion of females are classified as controllers (10.7% of females versus 7.4% of males). The mean spVL was ~0.36 logs lower in females as compared with males (Supplemental Figure 1). Of note, some of the cohorts specifically enrolled controller phenotypes, so these data do not reflect a random population sample.

### The impact of autosomal and chromosome X single genetic variation on spVL and HIV controller status stratified by sex

#### Sex-stratified association analysis of autosomal single genetic variation with spVL and with HIV control.

**Figure 1:**
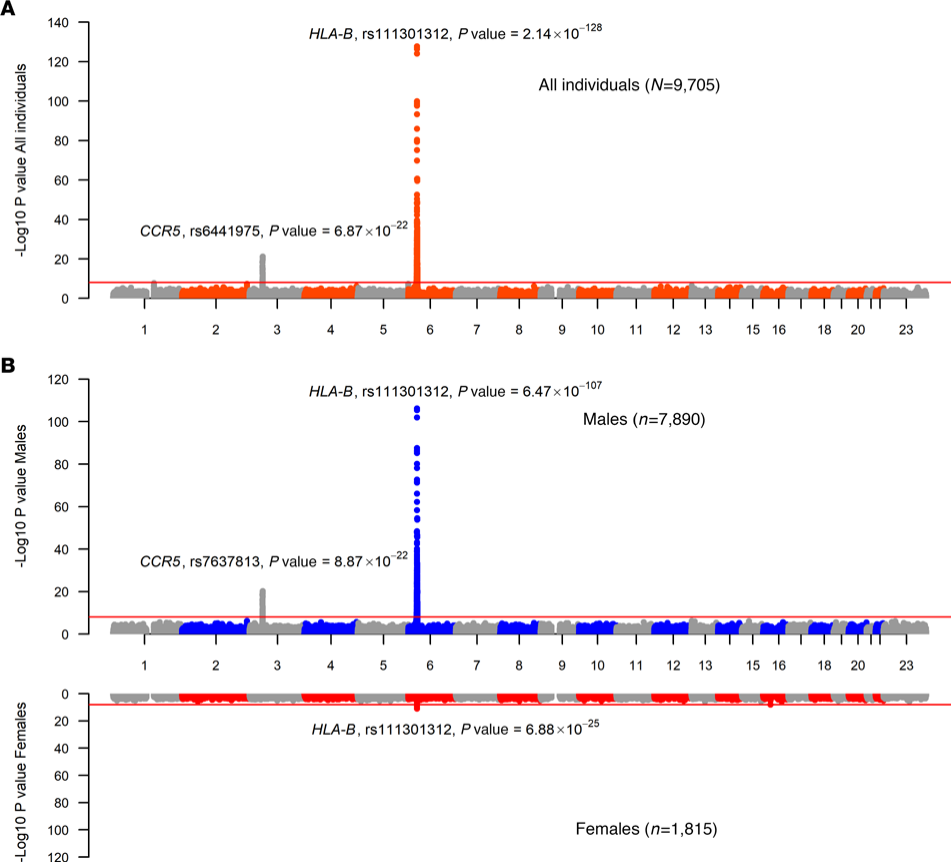
Manhattan plots of genome-wide association results between single genetic variants (MAF > 0.01) and HIV spVL. (**A** and **B**) Association was tested using linear mixed models the full sample, males only, and females only. Each dot indicates a genetic variant tested for association with spVL ordered by their physical position in the genome (*x* axis) and strength of association (–log_10_[*P* value], *y* axis). Coordinates are based on GRCh38/hg38. The red line indicates statistical significance accounting for multiple comparisons (*P* = 8.3 × 10^–9^). The signals on chromosome 3 and chromosome 6 correspond to the *CCR5* and *HLA* class I regions, respectively — both previously identified as impacting spVL.

After quality control and genome-wide genotype imputation (methods), we tested ~6.2 million common (minor allele frequency [MAF] > 0.01) autosomal single nucleotide polymorphisms (SNPs) for association with spVL and HIV control in the full sample and in males and females separately using linear mixed models (Figure 1 and Supplemental Figure 2). In both the full sample (Figure 1A and Supplemental Figure 2A) and in the analysis of males only, we observed 2 loci strongly associated with spVL and HIV control on chr3p21.31 and chr6p21.33 corresponding to the known genetic effects on viral load conferred by *CCR5* and the major histocompatibility complex, class I, B (*HLA-B*) genes, respectively. In the analysis of females only, we again observed a strong signal of association in the *HLA* region on chr6p21.33 but the chr3p21.31 region did not surpass statistical significance in this study (*P* < 8.3 × 10^–9^; Figure 1B and Supplemental Figure 2B).

Association statistics for top-associated variants per locus in each analysis are shown in Table 1. The direction of the effect and magnitude of the top-associated variants in the 2 loci are consistent in the full sample and each analyzed sex group. The top spVL-associated SNP in the *HLA* region in the full sample, and in the analysis of females only, was rs111301312, where the T allele conferred a 0.82–0.87 reduction in log_10_ (HIV RNA copies/mL of plasma) depending on the analyzed group (*P_full_* *_sample_* = 2.14 × 10^–128^, *P_males_* = 6.47 × 10^–107^, *P_females_* = 6.88 × 10^–25^). In the male-only analysis, the top-associated chromosome 6 variant was rs112515516 (*P_males_* = 9.36 × 10^–128^), which is in strong linkage disequilibrium (LD) with rs111301312 (*r^2^* = 0.99). Both SNPs reside in close proximity to *HLA-B*, the gene with the strongest described impact on spVL (21). The top-associated variants identified at the chromosome 3 locus in the full sample (rs6441975, *P_full_* *_sample_* = 6.87 × 10^–22^) and in males only (rs7637813, *P_males_* = 8.87 × 10^–22^) are also in strong LD with each other (*r^2^* = 0.91) and flank the *CCR5* gene, known to influence both HIV susceptibility and disease progression. This locus was not associated in females. Outside of these known loci, we did not observe statistically significant associations with spVL or HIV control in the full sample or sex-stratified analysis, suggesting no strong sex-specific autosomal effects at the single-variant level.

#### Assessment of heterogeneity of the SNP-based association at the CCR5 locus with spVL between sexes.

To explore whether the lack of association between spVL and markers on the *CCR5* locus in the female-only analysis was related to sample size or biological differences in the role of CCR5, we next analyzed the heterogeneity of association at the *CCR5* locus between the sexes. CCR5 expression on CD4 T cells has been reported to be impacted by exposure to sex steroid hormones (22) and may introduce more variability into associations with viral load phenotype. We have previously shown that the *CCR5* locus harbors at least 2 independent genetic variants (18), including the CCR5delta32 deletion and rs1015164, the latter of which is an expression quantitative trait locus (eQTL) influencing expression of a CCR5 antisense (CCR5-AS) long noncoding RNA that regulates CCR5 expression (23). We tested for evidence of heterogeneity in effect size (β) between males and females at the top-associated *CCR5* variant in the full sample (rs6441975), the top-associated variant in males only (rs7637813), a variant in strong LD with CCR5delta32 (rs113341849), and the CCR5-AS variant (rs1015164). Although we observed the largest difference in effect size at the CCR5delta32 proxy variant (β *_males_* = –0.311, β *_females_* = –0.143), there was no statistically significant heterogeneity observed at any of the *CCR5* variants tested (Supplemental Table 2). The concordance of the β values and lack of heterogeneity suggests that the lack of signal on *CCR5* locus in the females is likely due to the smaller sample size or ancestral balance compared with the male sample, rather than a biological difference in the impact of *CCR5* gene.

#### Sex-stratified association analysis of X chromosome genetic variation with spVL and HIV control status.

After applying quality control, we analyzed 6,953 individuals with available X chromosome genotype and spVL data across cohorts. We tested 126,135 high-frequency (MAF > 0.01) genetic variants in chromosome X for association in the full sample and in males and females separately. In both the full sample (Figure 1A) and in the stratified analysis of males and females only (Figure 1B), no X chromosome markers reached statistical significance, suggesting an absence of large effect size variants on the X chromosome for spVL. Similar results were found for HIV control (Supplemental Results and Supplemental Figure 2).

#### Gene-based analysis of sex-specific associations with spVL and HIV control status.

**Figure 2:**
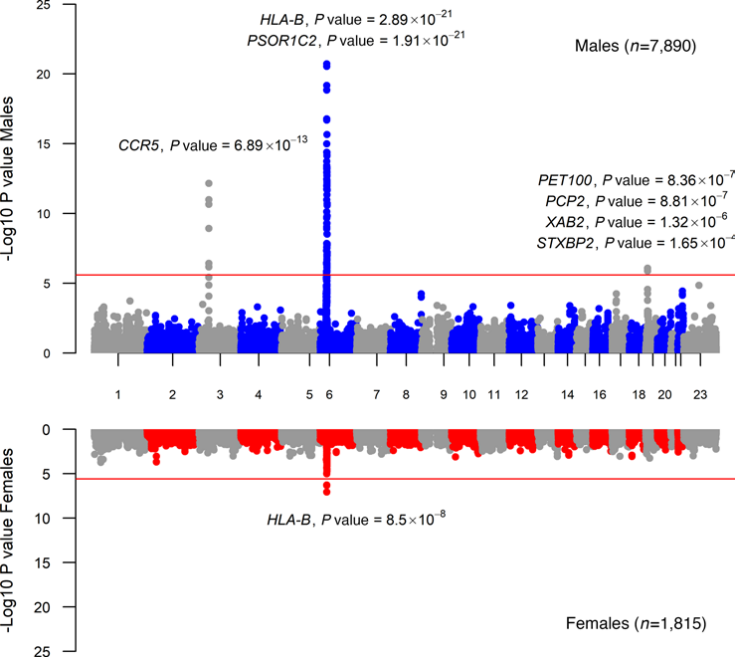
Miami plots of gene-based association analysis with HIV spVL in males and females. SNPs were assigned to the genes obtained from Ensembl build 85 (all genes). Each dot indicates a gene tested for association with spVL ordered by their physical position in the genome (*x* axis) and strength of association (–log_10_[*P* value], *y* axis) as calculated by MAGMA. Coordinates are based on GRCh37/hg19. The red line indicates statistical significance accounting for multiple comparisons (*P* = 2.56 × 10^–6^). *CCR5*, C-C motif chemokine receptor 5; *PSORS1C2*, psoriasis susceptibility 1 candidate 2; *HLA-B*, major histocompatibility complex, class I, B; *PET100*, PET100 cytochrome c oxidase chaperone; *XAB2*, XPA binding protein 2; *STXBP2*, syntaxin binding protein 2; *PCP2*, Purkinje cell protein 2.

We next used a gene-based approach, including both autosomes and the X chromosome, to assess whether the combined impact of individual variants within/near genes were significantly associated with spVL or HIV control status in a sex-specific manner. Markers from the GWAS were assigned to 19,495 protein coding genes based on proximity, and genes were tested for association using Multi-marker Analysis of GenoMic Annotation algorithm (MAGMA) (24). We observed 112 genes that were associated with spVL in the full sample, 97 of which were significantly associated in the male-only analysis (Supplemental Table 3), with the majority (94 genes) located in known associated regions and 3 genes uniquely associated in males on chr19p13.2 corresponding to PET100 cytochrome c oxidase chaperone (*PET100*, *P_males_* = 8.36 × 10^–7^), Purkinje cell protein 2 (*PCP2*, *P_males_* = 8.8 × 10^–7^), and XPA binding protein 2 (*XAB2, P_males_* = 1.32 × 10^–6^). In the same region, we observed a suggestive association with the gene syntaxin binding protein 2 (*STXBP2, P_males_* = 1.65 × 10^–4^, Figure 2 and Supplemental Table 3. The gene-based association was driven by 29 markers located on GRCh37/hg19:chr19:7697779-7713507 with *P_males_* < 5 × 10^–5^ with a consistent negative effect on spVL (β *_males_* = – 0.07, SEM *_males_ =* 0.018), Supplemental Table 4. This region is ~46 kb downstream of *PET100*, and it harbors the promoter and the entire coding region for *STXBP2* and promoters and part of coding region of *PCP2*. Additionally, our functional mapping analysis indicated that the top SNP in this region (rs885433, β *_males_* = –0.083, SEM *_males_* = 0.018, *P_males_* = 2.52 × 10^–6^) is an eQTL for *XAB2*, *STXB2*, and *PCP2* in multiple tissues of GTEx. Markers in this region in females had a smaller effect size and were not significantly associated in females (for example, rs885433, β *_females_* = –0.011, SEM *_females_* = 0.78, *P_females_* = 0.041; Supplemental Table 4).

Only 3 genes, all located on chromosome 6, were significantly associated with spVL in females. The top gene associated in females was *HLA-B* (*P_females_* = 8.5 × 10^–8^), with MHC class I polypeptide–related sequence A (*MICA*) and *MICB* also showing significant associations (Supplemental Table 3). Even though the direction of the effect was similar in females and in males for gene-mapped markers in the chr19p13.2 locus, *P* values did not indicate significance, and no genes were associated in females (Supplemental Table 4). Similar results in chromosomes 3 and 6 were observed in the analysis of HIV control (Supplemental Table 5 and Supplemental Figure 3). In summary, gene-based analysis indicated that all genes associated with spVL in both sexes on chromosomes 6 and 3 were also associated in all individuals, but genes on the chromosome 19 region were only associated in males (Figure 2 and Supplemental Table 3).

### Analysis of variants with a differential effect on spVL between sexes

#### SNP-based analysis of markers with differential effect on spVL between sexes.

**Figure 3:**
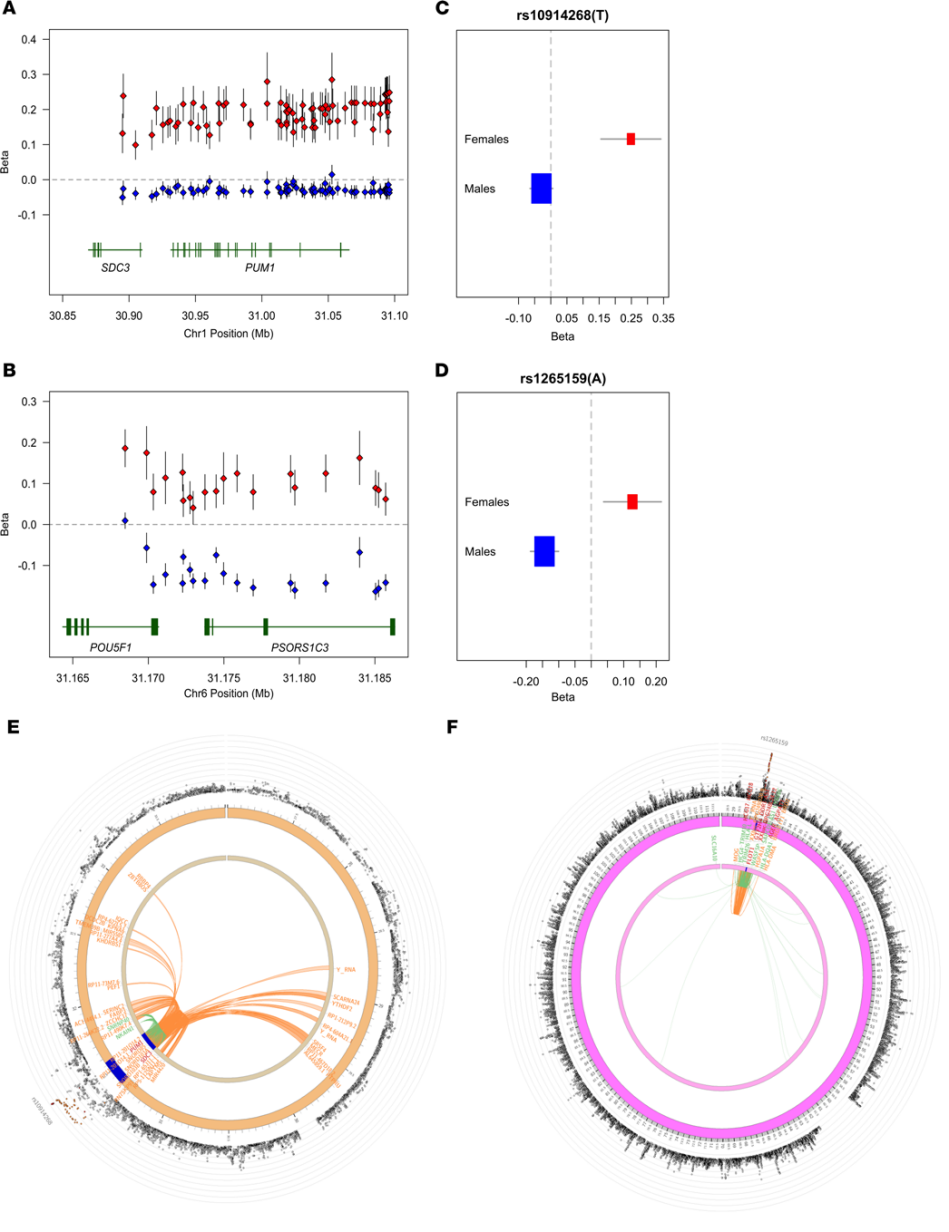
Variants on chr1p35.2 and chr6p21.33 regions showing strong heterogeneity in direction and size of effect on HIV spVL in males compared with females. (**A** and **B**) Effect size (β) point estimates (diamonds) and standard error (whiskers) for variants showing strong differences between females (red) and males (blue). The *x* axis shows genomic positions (GRChg38/hg38) in mega-bases. The *y* axis denotes betas from linear regression models in units of HIV RNA copies/mL of plasma. Protein coding genes in the region are included at the bottom in green. (**C** and **D**) Effect sizes (beta) and standard error of the variant with the most significantly different effects between males and females in each region. The rs number and effect allele are listed above each plot. *P* values were calculated using 2-tailed Student’s *t* test. (**E** and **F**) Circos plots of chromatin interactions and eQTLs for chr1p35.2 and chr6p21.33 loci, respectively. In the most outer layer is a Manhattan plot, showing only SNPs with *P* < 0.05. SNPs in genomic risk loci are color coded as a function of their maximum *r^2^* to the 1 of the independent significant SNPs in the locus, as follows: red (*r^2^* > 0.8), orange (*r^2^* > 0.6), green (*r^2^* > 0.4), and blue (*r^2^* > 0.2). SNPs that are not in LD with any of the independent significant SNPs (with *r^2^* ≤ 0.2) are gray. The rs number of the top SNPs in each risk locus are displayed in the most outer layer. The *y* axis (represented by the expanding rings around the central circular locus map) is ranged between 0 to the maximum –log_10_(*P* value) of the SNPs. The second layer displays the chromosome ring, where genomic risk loci are highlighted in blue. Inside this ring are the acronyms of the genes mapped by chromatin interactions or eQTLs. Links to genes and genes mapped only by chromatin interactions or only by eQTLs are colored orange or green. Links for genes and genes mapped by both are colored in red. *PSORS1C2*, psoriasis susceptibility 1 candidate 2; *SDC3*, syndecan 3; *PUM1*, pumilio RNA binding family member 1.

To assess whether there was evidence of broad-scale heterogeneity in the impact of genetic variants on spVL between the sexes, we compared the effects of the sex-stratified GWASs using 2-tailed Student’s *t* test. We observed 2 loci with a significant differential effect, with several variants with a positive (spVL increasing) effect observed in females compared with a negative (spVL decreasing) or neutral effect in males. The first locus is on chr1p35.2 (Figure 3) and included 54 SNPs in LD in the region GRCh38/hg38: chr1:31092908-31096084, harboring the genes syndecan 3 (*SDC3,* ~ 187 kb from top SNP) and pumilio RNA binding family member 1 (*PUM1,* ~ 30 kb from top SNP). Allele T of rs10914268 (G>T, *P* = 1.93 × 10^–8^) has a suggestive association with a positive effect on spVL in females but has no significant effect in males (β *_females_*: 0.25, SEM *_females_* = 0.047, *P_females_* = 2.39 × 10^–7^; β *_males_*: –0.029, SEM*_males_* = 0.018, *P_males_* = 0.106; Supplemental Table 6). The second locus is chr6p21.33 (rs1265159:G>A, *P* = 3.26 × 10^–8^), including 95 SNPs in LD in the genomic region chr6:31172270-31198575. This is within the MHC region and harbors genes such as psoriasis susceptibility 1 candidate 1 (*PSORS1C1*) and *PSORS1C2* (with top SNPs located 33 kb downstream of the gene), transcription factor 19 (TCF19, top SNP located ~8 kb downstream of the gene), and corneodesmosin (CDSN, top SNP located 51 kb upstream of the gene). Allele A of the top SNP, rs1265159 (G>A, *P* = 3.26 × 10^–8^) has a suggestive association with a positive effect on spVL in females, but it has a significant association and negative effect in males (β *_females_*: 0.127, SEM *_females_* = 0.05, *P_females_* = 0.0052; β *_males_*: -0.144, SEM *_males_* = 0.022, *P_males_* = 8.10 × 10^–11^; Supplemental Table 6). There were no markers associated with major effects in males compared with females.

#### SNP-based analysis of markers with differential effect on HIV control between sexes.

**Figure 4:**
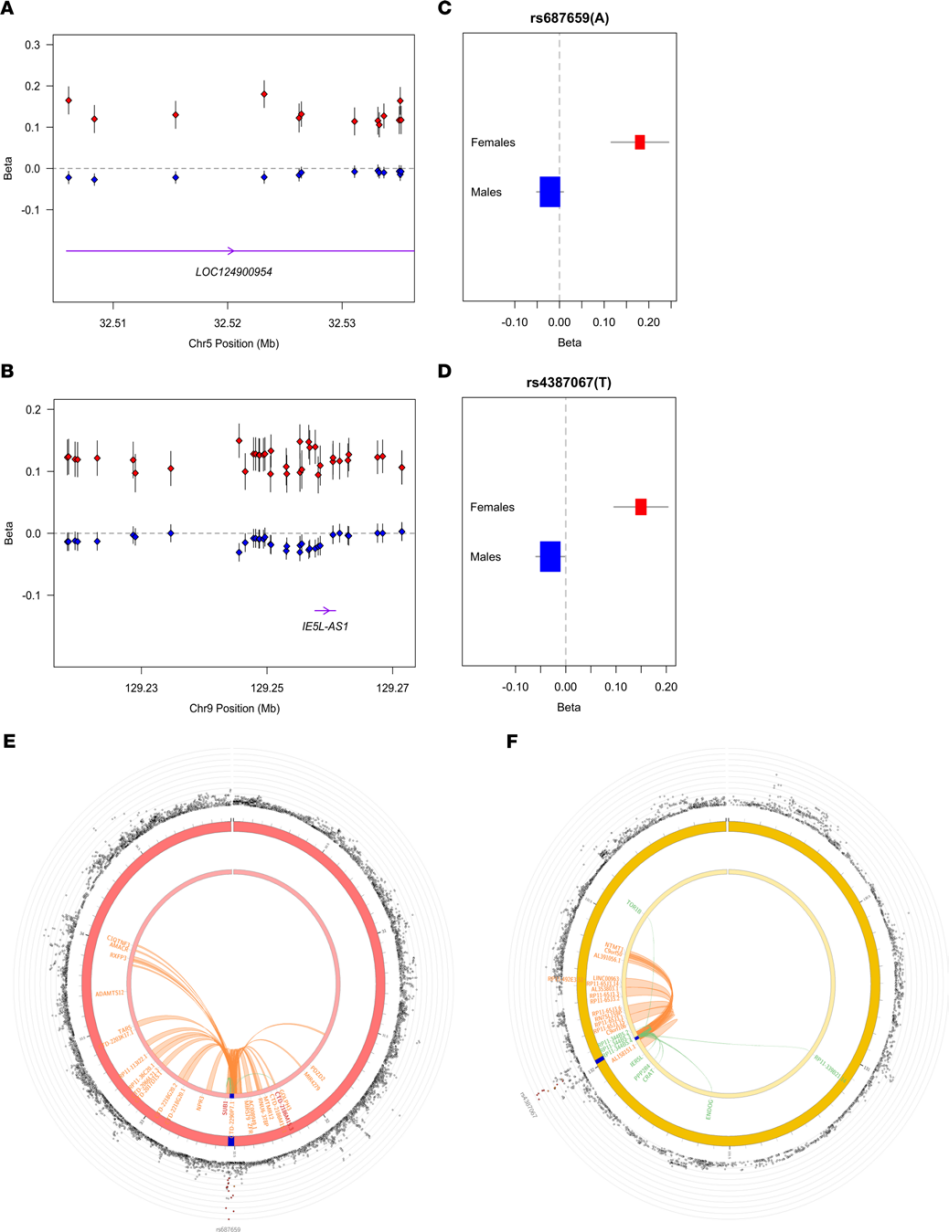
Variants on chr5p13.3 and chr9q34.11 regions showing strong heterogeneity in direction and size of effect on HIV control in males compared with females. (**A** and **B**) Effect size (β) point estimates (diamonds) and standard error (whiskers) for variants showing strong differences between females (red) and males (blue). The *x* axis shows genomic positions (GRCh38/hg38) in mega-bases. The *y* axis denotes betas from logistic regression models. Noncoding RNAs in the region are included at the bottom, in purple. (**C** and **D**) display effect sizes (β) and standard error of the variant with the most significantly different effects between males and females in each region. The variant rs number and effect allele are listed above each plot. *P* values were calculated using 2-tailed Student’s *t* test. (**E** and **F**) Circos plots of chromatin interactions and eQTLs for chr 5p13.3 and chr9q34.1 loci, respectively. In the most outer layer is a Manhattan plot, showing only SNPs with *P* < 0.05. SNPs in genomic risk loci are color coded as a function of their maximum *r^2^* to the 1 of the independent significant SNPs in the locus, as follows: red (*r^2^* > 0.8), orange (*r^2^* > 0.6), green (*r^2^* > 0.4), and blue (*r^2^* > 0.2). SNPs that are not in LD with any of the independent significant SNPs (with *r^2^* ≤ 0.2) are gray. The rs number of the top SNPs in each risk locus are displayed in the most outer layer. The *y* axis (represented by the expanding rings around the central circular locus map) ranged between 0 to the maximum –log_10_(*P* value) of the SNPs. The second layer displays the chromosome ring where genomic risk loci are highlighted in blue. Inside this ring are the acronyms of the genes mapped by chromatin interactions or eQTLs. Links to genes and genes mapped only by chromatin interactions or only by eQTLs are colored orange or green. Links for genes and genes mapped by both are colored in red. *SUB1*, SUB1 regulator of transcription; *PTPA*, protein phosphatase 2 phosphatase activator; *IER5L*, immediate early response 5 like; *AL158151.3*, protein AL158151.3.

We applied the same analysis to HIV control, comparing effects of loci on HIV control for each sex and identified 2 loci, both with a greater magnitude of effect in females compared with males. The first locus is in chr9q34.11, 15 kb downstream from the gene coding for the protein *AL158151.3*, protein phosphatase 2 phosphatase activator (*PTPA,* ~96 kb), and ~67 kb upstream of the gene immediate early response 5 like (*IER5L*). The top variant rs4387067 (C>T, *P* = 2.07 × 10^–9^) conferred an independent signal in this region with 16 SNPs in LD with the top variant. This marker is suggestively associated in females and allele T has positive effect on HIV control in females (OR*_females_* = 1.16, *P_females_* = 1.18 × 10^–5^) and a negative effect in males (OR*_females_* = 0.97, *P_males_* = 0.03, Supplemental Table 7). The second locus is in chr5p13.3, approximately 80 kb upstream from the gene SUB1 regulator of transcription (*SUB1*). The top variant is rs687659 (T>A, *P* = 1.02 × 10^–8^) conferring an independent signal with 20 SNPs in LD with the top variant. This marker is suggestively associated in females, with the A allele having a positive effect on HIV control in this group (OR*_females_* = 1.20, *P_females_* = 1.2 × 10^–5^) and a negative effect in males (OR*_males_* = 0.98, *P_males_* = 0.016), as shown in Figure 4 and Supplemental Table 7.

#### Functional mapping of markers with differential effect on spVL and HIV control between sexes.

We next explored potential functionality of the SNPs with sex-differential effects on spVL and HIV control. In the chr1p35 region, 54 candidate SNPs were classified *cis*-eQTL and likely affect expression of *SDC3* (minimum eQTL *P* = 3.48 × 10^–24^) and *PUM1* (minimum eQTL *P* = 2.12 × 10^–24^) and other genes in the region including sodium/potassium transporting ATPase interacting 1 (*NKAIN1*) and small nuclear ribonucleoprotein U5 subunit 40 (*SNRNP40*). The top SNPs with differential effect in this locus are located in a region that has interaction with promoters/enhancers in nearby genes as well as remote genes in the same chromosome (Figure 3 and Supplemental Table 5). Similarly, in the chr6p21.33 locus, top SNPs are eQTLs for *PSORS1C2*, *PSORS1C1*, and *HLA-B* and for other genes located in the same chromosome (Figure 3 and Supplemental Table 8). The same variants are *trans*-eQTL of genes located in other chromosomes (data not shown).

Top markers with sex differential effect for HIV control in chr5p13.3 were cataloged as *cis-*eQTL SNPs (*P* = 3.6 × 10^–7^) for several genes in the region, including *SUB1* and *IER5L,* and were located in regions with interaction with enhancer promoters of other genes in the same chromosome (Figure 4). In the chr9q34.11, top SNPs located in the *AL158151.1* gene were also identified as eQTL for *RP11-344B5.4*, *RP11-344B5.3*, and *RP11-344B5.2* (Figure 4). Other genes in the region were identified as related with both loci, as described in Supplemental Table 9.

## Discussion

Despite the consistent and substantial differences between males and females in HIV spVL (1) and in the frequency of spontaneous HIV control (5–7), the mechanisms for these differences are incompletely defined. Understanding the host factors that determine HIV disease pathogenesis and the variation across populations by age, sex, and genomic features has the potential to inform efforts at preventive vaccination, therapeutic, and curative interventions. In this study, we used the largest available collection of genomic data to explore whether there are sex-specific autosomal or X chromosome variants that have an impact on either spVL or HIV controller status. We compared the sex-specific genetic architecture of both traits in both stratified and combined analyses. We identified sex-shared associations at the single-variant level, sex-specific associations at the gene-based level, and genetic variants with significant differential effects between the sexes. Taken together, these data suggest that, apart from the known determinants of large effect, additional genomic features contribute to spVL and HIV controller status, and some of these features have sex specificity.

At the single-variant level, we confirmed the presence of known signals associated with spVL and viral control in the MHC region in both sexes (with a smaller strength of association in females) and the *CCR5* region in males but not in females. The small area of association around the *HLA* locus observed in females, in contrast to a region of several megabases identified in males, and heterogeneity analysis of the *CCR5* region — which did not identify significant sex differences — suggest that the female-only analysis is limited by sample size. A sensitivity analysis with a downsized male sample (*n* = 1,815), identical to the female sample size, showed a decrease in the association of the *CCR5* region (no longer reaching significance to GWAS level, rs4683219, *P_males_* *_(downsized)_* = 3.87×10^–7^) and the MHC region (rs112515516, *P_males_* *_(downsized)_* = 1.33 × 10^–32^), again consistent with decreased power in our female analysis. This leaves open the question of whether novel associations in females only were missed by this analysis.

Our gene-based association approach, which can amplify signals from multiple, correlated SNPs in a single gene region, identified a male-only association in a cluster of genes including *XAB2*, *PCP2*, *PET100*, and *STXBP2* on chromosome 19. These genes include a few of potential functional significance in HIV infection. *STXBP2,* although not the top-associated gene within our analytic parameters, is immunoactive. It plays a role in vesicle trafficking, and mutations limit release of cytotoxic granules by NK cells and cytotoxic CD8^+^ T cells, leading to a form of familial hemophagocytic lymphohistocytosis (25). There is evidence that NK cell function affects HIV pathogenesis (26), but little data are available regarding whether NK cell function would be predicted to have a sex-differential association. Of note, distinct from these proposed immune roles, in a Jurkat model system, *STXBP2* was among the genes identified with a role in HIV replication, and knockdown led to a reduction of HIV DNA reverse transcription (27). The precise mechanism of this cell-intrinsic mechanism of restricting HIV replication is unclear. *XAB2*, another gene in this region, was identified in an siRNA screen in the 293T cell model of HIV infection as a host factor with a proposed role in DNA repair during reverse transcription, blocking replication through impacts on viral RNA kinetics or DNA stability (28). *XBA2* was also identified in a CRISPR-Cas–KO screen of host factors regulating expression of HIV RNA transcripts (29). The exclusive association of this region in males only and not in the full data set and the smaller effect size observed in females suggests that there may be sex-specific factors that intersect with the roles of these genes in HIV replication.

In summary, after considering potential lack of power in our female sample, we can suggest that the genetic architecture of the HIV viral load and HIV viremic control has shared elements between the sexes, predominantly in the *HLA* locus and *CCR5* genes but also some particular genetic risk factors only detected in males with our current sample size. It is possible that there are female-specific signals not detectable with the female sample size; however, as noted above, several of these signals increase in strength in the male-only analysis, implying that the female signal is not concordant. This highlights the gap in available data, as this is the largest genomic association study and still does not appear to have adequate power to explore all associations in females, who are statistically more likely to achieve viral controller status (5–7) and have lower spVL (1). Our analysis is also limited primarily to European and African ancestry, with minimal inclusion of other ancestry backgrounds.

Our analysis exploring concordance or discordance of genetic associations between the sexes suggests that there are sex-intrinsic impacts in some of the shared immune-related genes in the *HLA* region that confer a larger effect in females versus males. Additionally, this analysis highlighted other regions with plausible or previously demonstrated roles in HIV replication or immune function that have a sex-discordant association with HIV spVL (*SDC3*, *PUM1*) and/or viral control (*SUB1, AL158151.3*). Among these genes, *SDC3* is a DC-specific proteoglycan that interacts with the HIV-1 envelope glycoprotein gp120, facilitating DC infection and transmission of infection of T cells (30). In one study, monocyte-derived DCs from HIV controllers expressed higher levels of SDC3, prompting the hypothesis that enhanced viral particle capture, combined with reduced susceptibility to infection, may facilitate DC priming of T cell responses (31). *PUM1* is regulator of innate immune gene expression via initial suppression of *LGP2* (laboratory of genetics and physiology 2, also known as *DHX58*) expression, followed by a rise in LGP2 with subsequent expression of innate sensing and IFN-stimulated genes, including melanoma differentiation-associated gene 5 (*MDA5*), retinoic acid–inducible gene I (*RIG-I)*, stimulator of IFN genes (*STING)*, IFN-β (*IFNb),* and IFN-induced protein with tetratricopeptide repeats 1 (*IFIT1*) (32). Given the known differences in baseline and stimulated expression of IFN pathways in males and females, this may be a source of sex specificity in immune response. *SUB1,* associated with a differential impact on viral control, was identified as part of the same DNA damage repair cluster that includes *XBA2* in the siRNA 293T cell screen of host factors impacting HIV, with a putative role in DNA repair during reverse transcription (28). Separately, *SUB1* was identified as a host protein interacting with the HIV transactivator protein tat (33). Less is known about the other putative genes with a differential effect on HIV control (*AL158151.3*). We also queried our data for *TLR7* an X chromosome gene with polymorphisms suggested to have a role in HIV pathogenesis in prior small cohort studies (34, 35). We had adequate coverage of this region but did not observe any significant associations for markers in this locus.

Our findings of gene-based associations with HIV outcomes in the male cohort are intriguing and should be validated in an independent data set that includes a more genetically diverse populations and a more balanced sex distribution. Many of the genes in the associated regions are involved in immune processes, have been identified in screens of host factors relevant to HIV replication and/or harbor causal variants with high penetrance for diseases with an unequivocally immunological basis. These features of the regions of association reinforce their potential significance. Inclusion of the X chromosome and stratified analyses may identify genomic factors that have phenotypic implications only in 1 sex. Nonchromosomal factors including sex steroid hormone exposure and sex-specific epigenetic regulation of gene expression should also be investigated. Taken together, these data will allow further mechanistic definition of the determinants of HIV pathogenesis across diverse populations.

## Methods

### Participants, genotyping, and quality control

All participants were adults with HIV-1 infection. Genome-wide autosomal genotypes and clinical data were obtained on a total of 9,705 PLWH from the ICGH (19). Genotype data were generated on various platforms at local genotyping centers (Supplemental Table 1). Harmonized genotype quality control was performed on autosomal genetic variants for all samples as previously described (18). Briefly, ancestry was inferred by principal components (PC) analysis using EIGENSTRAT (36), using the 1000 Genomes Phase 3 v5 sample as a reference (37). Only samples matching reported ancestry with the 1000 Genomes European or African samples were included. Study participants were excluded based on the following criteria: identity-by-descent of > 0.125 (1 individual per pair was removed), missingness of > 2%, and inbreeding coefficients of < −0.1 or > 0.1. SNPs were removed based on missingness of > 5%, MAF of < 1%, or deviation from Hardy-Weinberg equilibrium of *P* < 1 × 10^−7^.

### Phenotype definitions

spVL was defined as the log_10_ of the average number of HIV-1 RNA copies/mL of plasma from at least 2 viral load measurements during the chronic phase of infection and prior to the initiation of ART, as previously described (38). We additionally used a binary definition of HIV spontaneous control in the subset of samples obtained from the International HIV Controllers Study and the AIDS Clinical Trials Group (Supplemental Table 1) (17). Briefly, HIV controllers were defined as having a minimum of 3 measurements of plasma viral load < 2,000 RNA copies/mL over at least a 1-year period prior to initiation of ART, consistent with the definition of control used in the original manuscript (17). Of note, this is distinct from elite suppressors or elite controllers who are more typically defined as having plasma viral load measurements below the limit of detection on clinical assays. HIV noncontrollers included individuals who did not meet these criteria and required ART initiation.

### Statistics

#### Imputation and association testing of autosomal genetic variants.

Per cohort, unobserved genetic autosomal variants were imputed using TOPMed and version R2 GRCh38 of the human genome reference panel following standard data preparation and imputation recommendation (39). After imputation, individual data sets were filtered, and variants with low frequency (MAF < 1%) and/or low imputation quality (*R*^2^ < 0.3) were excluded. Data sets were combined, and an intersecting set of variants (i.e., genotyped or imputed in all sets) were carried forward to association testing. The association between autosomal genetic variants and spVL was tested using linear mixed models implemented in Genome-wide Efficient Mixed Model Association (GEMMA) program (v0.98.1) (40) in the total sample and stratified by sex. We used this same procedure for the HIV controller/noncontroller analysis using the binary phenotype and generalized linear mixed models. To limit potential for false-positive association, we considered variants with *P* < 8.3 × 10^–9^ as significantly associated. LD between genetic variants was calculated using the LD link LDpair tool (41) using the 1000 Genomes project sample as reference (37). Genetic coordinates for this analysis are based on GRCh38/hg38.

#### Testing for sex-based heterogeneity at known loci.

To assess whether the effect of known associated loci in the C-C motif chemokine receptor 5 (*CCR5*) region varied by sex, we selected the top-associated variants in this locus per analysis, the eQTL rs1015164 and a proxy variant for CCR5delta32 (rs113341849). To identify the CCR5delta32 proxy variant, we calculated LD between variants in the *CCR5* region and sequence-based CCR5delta32 types in a subset of our sample with both data types available (*n* = 4,437). This analysis demonstrated that rs113341849 was the best proxy for CCR5delta32 (*r*^2^ = 0.96) in our sample. Differences in effect sizes were assessed by comparing the β values from the spVL analysis in males versus females using Woolf’s test for heterogeneity. *P* < 0.05 was considered statistically significant.

#### Genotyping, imputation, and association testing of chromosome X genetic variants.

Chromosome X genotype and clinical data were available for a total of 6,953 participants included in the autosomal analysis. X chromosome–specific harmonized genotype quality control was done using the XWAS v3.0 software protocol (42) by removing variants in the pseudo-autosomal regions and filtering out variants if they had significantly different MAF or missingness between male and female controls as well as variants that were not in Hardy-Weinberg equilibrium in females. Chromosome X unobserved genetic variants were imputed, filtered, selected for association, and tested using the methods described for autosomal variants. Association between chromosome X genetic variants and spVL was tested separately in each genetically determined ancestry group (European and African), stratified by sex to reduce inflation. Since this is the first association analysis to our knowledge of markers on the X chromosome in this multiancestry study, in addition to the sex-specific association, we evaluated the association in the complete population. Summary statistics of the associations on the X chromosome in each sex and ancestry group (4 data sets) were meta-analyzed using Stouffer’s method (43) as implemented in METAL (44) to produce a significance value for each SNP in the entire population. This method accommodates the possibility of differential effect size and direction between males and females and is not affected by assumptions about chromosome X inactivation or escape of inactivation in females. We used this same stratification for the binary HIV controller/noncontroller phenotype generalized linear mixed models in GEMMA v0.98.1 (40) and multiancestry metanalysis in the entire data set as described for the spVL phenotype.

#### Genome-wide comparison of single-variant genetic effect between the sexes.

We assessed whether each genetic variant had different effects in males and females using the following 2-tailed Student’s *t* test, as previously described (45–47).
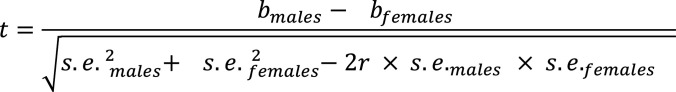


Where *b* is the estimated effect of the genetic variant for males and females for either spVL or HIV controller status, SEM is the standard error of the effect, and *r* is Spearman’s rank correlation between the sexes across all genetic variants for a given trait. For spVL, the correlation was 0.011 and for 0.015 for HIV control. To account for multiple testing, we considered a genome-wide significance cut-off of *P* < 5.0 × 10^–8^ for the lead SNP. To cluster our results into independent lead variants, we used the clump option in PLINK 1.9 (48, 49), including SNPs with a maximum *P* cutoff of 0.05 and an LD *r^2^* > 0.6 with the lead SNP as threshold to define independent significant SNPs using all populations of 1000 Genomes Project, Phase 3 as a reference group (37). We assigned 300 kb as the maximum distance between LD blocks to merge into a locus.

#### Gene-based analysis.

To increase power to detect associations, we evaluated gene-based units in place of SNPs by accounting for correlations among multiple SNPs within a gene. Genes enriched for variants associated with spVL or HIV control were identified using a gene-level association analysis (gene-based test) (24) implemented by MAGMA (24, 50) as part of the Functional Mapping and Annotation of Genome-Wide Association Studies suite (FUMA v 1.3.8) (51, 52). This gene analysis is based on a multiple-linear PC regression model (53), using an F-test to compute the gene *P* value. This model first projects the SNP matrix for a gene onto its PC, pruning away PC with very small eigenvalues, and it then uses those PC as predictors for the phenotype in the linear regression model. This improves power by removing redundant parameters and guarantees that the model is identifiable in the presence of highly collinear SNPs (24, 50).

Gene-level analyses were carried out using 2 approaches in each phenotype: (a) identification of associated genes with differential effects across the sexes and (β) identification of genes with sex specific associations. Hence, to obtain genes with a larger genetic effect in each of the sexes, we partitioned our 2-tailed single variant *P* values (*P_2T_*) from the genetic effect (β) comparison between the sexes into two 1-tailed *P* values. For genetic variants where *βfemales* > *βmales*, 1-tailed *P* values were calculated as:
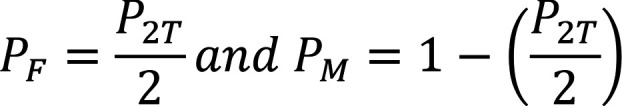


When the genetic effect was larger in males, the P values were calculated as:
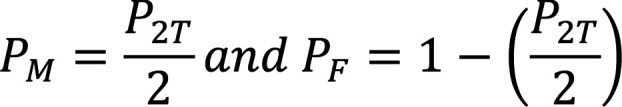


This process led to the creation of 2 additional distinct sets of *P* values for each phenotype, corresponding to sites where the genetic effect was significantly greater in males or females.

Each of these sets of *P* values (*P_2T_*, *P_M_*, and *P_F_*) was subsequently used to identify gene-level associations using MAGMA v1.08 (24). First, we annotated each gene (i.e., defined which SNPs were in the gene region), considering a region of 50 kb upstream and downstream of the gene. MAGMA was then run for each phenotype and each set of 1-tailed *P* values separately, considering the SNP-wise mean model. Input SNPs were mapped to 19,495 protein-coding genes annotated in The Genotype-Tissue Expression project (GTEx) v8. Genome wide significance was corrected by the number of genes and defined at *P* = 0.05/19,495 = 2.56 × 10^–6^, corrected by the number of genes (19,495). We adjusted the genetic coordinates for this analysis to GRCh37/hg19 to make the data compatible with the FUMA v 1.3.8 (51, 52) tool.

#### Functional gene mapping.

Functional gene mapping was performed by positional, eQTL, and chromatin interaction mapping based on all genes of Ensemble v92 (54) implemented in FUMA v1.3.8 (51, 52). For positional mapping, candidate SNPs (independent significant SNPs and their proxy SNPs) were mapped to genes located at a physical distance of 50 kb from the markers. For eQTL mapping, we paired candidate SNPs to genes located up to 1 Mb away and whose expression is likely affected by the SNPs (*cis*-eQTL) in the complete GTEx data set. eQTL results were limited to significant SNP-gene pairs that were defined as associated SNPs with an FDR < 0.05 in the GTEx v6, v7, and v8 data sets (55). For chromatin interaction mapping, we used built-in chromatin interaction data such as Hi-C of 21 tissue/cell types from GSE87112 (https://www.omicsdi.org/dataset/geo/GSE87112) (56), preprocessed enhancer-promoter and promoter-promoter interactions based on Hi-C data for adult and fetal human brain samples (57), Hi-C–based data from PsychENCODE (58), and enhancer and promoter regions obtained from the Roadmap Epigenomics Projects for 111 epigenomes (59). We mapped candidate SNPs to chromatin interacting regions, and we mapped those regions to genes whose promoters overlapped with them. We defined a promoter regions as 250 bp upstream and 50 bp downstream of the transcription starting site. An FDR < 1 × 10^–6^ was used as threshold for a significant chromatin interaction. Coordinates for this analysis are based on GRCh37/hg19.

#### Study approval.

Written informed consent for genetic testing was obtained by each study from all participants. Ethical approval was obtained from IRBs for each of the contributing centers (Supplemental Table 1).

## Author contributions

EPS and PJM contributed to the acquisition of funding, conception, design of the study, interpretation of results, and writing of the manuscript. PD and JF contributed to interpretation of results and revision and writing of the manuscript. JFT and CV contributed to the data quality control, statistical analysis of autosomes and X chromosome, interpretation of results, and preparation and writing of the manuscript. CV contributed to the gene-based, sex differential analyses and final preparation of the manuscript. ICGH contributed data to this project.

## Supplementary Material

Supplemental data

Supplemental table 3

Supplemental table 4

Supplemental table 5

Supplemental table 6

Supplemental table 7

Supplemental table 8

Supplemental table 9

## Figures and Tables

**Table 1:**
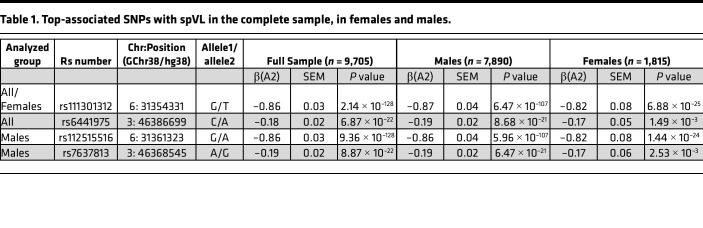
Top-associated SNPs with spVL in the complete sample, in females and males.

## Version 1 04/25/2023

In-Press Preview

## Version 2 06/08/2023

Electronic publication
