# Supplementary material for: Multiancestry sex-stratified genomic associations with HIV viral load and controller status from the ICGH
Source: JCI Insight. 2023 Jun 8;8(11):e170068. doi: 10.1172/jci.insight.170068 (PMC10393222; doi:10.1172/jci.insight.170068)
Supplement: Supplemental data [file jciinsight-8-170068-s238.pdf]

Multi-ancestry sex-stratified genomic associations with HIV viral load and controller status from the ICGH

Supplementary Results

<u>Results</u>

The impact of autosomal and chromosome X single genetic variation on HIV controller status stratified by sex.

<u>*Sex-stratified association analysis of autosomal single genetic variation with HIV control status*</u>

Given the known imbalance in HIV controller status between males and females (1-3), we assessed the impact of autosomal variation on HIV viremic control. We used the subset of our sample contributed by the International HIV Controllers Study and the AIDS Clinical Trials group (n=2,831) used in a previous GWAS of HIV control (4). We tested for association between autosomal genetic variation and a binary HIV controller/non-controller phenotype using linear mixed models in the full sample (n=778 controllers, 2,053 non-controllers, **Supplementary Figure S2A**), males only (n=584 controllers, 1704 non-controllers) and females only (n=194 controllers, 349 non-controllers, **Supplementary Figure S2B**). Consistent with the spVL analysis, we observed strong signals of association in the *HLA* region in all analyzed groups, significant associations in the *CCR5* region in the total sample and in males only and no significant associations outside of these loci in the sex-stratified analysis.

<u>*Sex-stratified association analysis of X chromosome genetic variation with HIV control status*</u>

After applying quality control, we analyzed 5,907 individuals with a controller phenotype in the total sample corresponding to 5,291 males (controllers=737, non-controllers=4554) and 616

females (controllers=227, non-controlers=389). No sex chromosome markers reached statistical significance for association with controller status in the full sample (**Supplementary Figure S2A**) and in males and females (**Supplementary Figure S2B**) separately.

References

1. Price MA, Rida W, Kilembe W, et al. Control of the HIV-1 Load Varies by Viral Subtype in a Large Cohort of African Adults With Incident HIV-1 Infection. *J Infect Dis*. 2019;220(3):432-441.

2. Madec Y, Boufassa F, Porter K, Meyer L, CASCADE Collaboration. Spontaneous control of viral load and CD4 cell count progression among HIV-1 seroconverters. *AIDS*. 2005;19(17):2001-2007.

3. Yang OO, Cumberland WG, Escobar R, Liao D, Chew KW. Demographics and natural history of HIV-1-infected spontaneous controllers of viremia. *AIDS*. 2017;31(8):1091-1098.

4. International HIV Controllers Study, Pereyra F, Jia X, et al. The major genetic determinants of HIV-1 control affect HLA class I peptide presentation. *Science*. 2010;330(6010):1551-1557.

Supplementary Tables and Figures

Cohort	Analysis of Autosomes (N=9,705)		Analysis of X chromosome(N=6,953)		Genetic Ancestry Group	Genotyping Platform
	n	% Female	n	% Female		
The International HIV Controllers Study & The AIDS Clinical Trials Group	2,824	19.2	2,019	14.3	EUR/AA	Illumina 550, Illumina 1M
ALIVE, MHGDS, MHCS, DCG	1,356	5.8	1,328	4.2	EUR/AA	Affymetrix 6.0
EuroCHAVI	1304	25.1	1,304	25.1	EUR	Illumina 650, Illumina 1M
The Multicenter AIDS Cohort Study	1117	0.0	1,117	0.0	EUR/AA	Illumina 1M
Urban Health Study: Genetics Cohort	769	24.6	0.0	0.0	EUR/AA	Illumina 650
The nonprogressor Genomics of Resistance to Immunodeficiency Virus Study & The ANRS PRIMO cohort	581	12.6	0.0	0.0	EUR	Illumina 300
CHAVI	515	4.5	0.0	0.0	EUR/AA	Illumina 1M
The Amsterdam Cohort Studies on HIV infection and AIDS	384	7.8	382	7.8	EUR	Illumina 300
The Swiss HIV Cohort Study	340	67.9	340	67.9	AFR	Illumina H3A African array
The International AIDS Vaccine Initiative	242	40.5	463	38.8	AFR	Illumina 1M
The Pumwani Sex Workers Cohort, University of Nairobi	147	100.0	0.0	0.0	AFR	Affymetrix 5.0
Wellcome Trust Sanger Institute	126	61.9	0.0	0.0	AFR	Illumina 1M

Supplementary Table S1. Characteristics of the individuals included in the analysis of autosomes and X chromosomes and distribution by cohort, sex, ancestry group and genotyping platforms. Acronyms: ALIVE: The AIDS Linked to the IntraVenous Experience Cohort; MHGDS: The Multicenter Hemophilia Growth and Development Study; MHCS: Multicenter Hemophilia Cohort Studies; DCG: the D.C. Gays cohort; EuroCHAVI: Center for HIV/AIDS Vaccine Immunology ;CHAVI: Center for HIV/AIDS Vaccine Immunology.

**Rs Number**	**Allele 1/ Allele2**	**Males (n =7,890)**		**Females (n=1,815)**		**P value Heterogeneity**	**Variant Description**
		***b* (A2)**	**s.e**	***b* (A2)**	**s.e.**		
**rs6441975**	C/A	-0.187	0.020	-0.172	0.054	0.794	Top *CCR5* variant in full sample
**rs7637813**	A/G	-0.186	0.019	-0.167	0.055	0.744	Top *CCR5* variant in males
**rs113341849**	A/G	-0.311	0.035	-0.143	0.115	0.162	*CCR5*d32 proxy
**rs1015164**	G/A	-0.185	0.020	-0.173	0.074	0.876	*CCR5*-AS eQTL

Supplementary Table S2. Analysis of heterogeneity in effect size (*b*) between males and females at the top associated *CCR5* variant in the full sample (rs6441975), the top associated variant in males only (rs7637813), a variant in strong LD with CCR5delta32 (rs113341849) and the CCR5-AS variant (rs1015164). Acronyms: *b*: beta; s.e.: standard error.

Supplementary Table S3. Genes associated with HIV spVL in the full sample, males only and females only analyses (excel file).

Supplementary Table S4. Markers located on chr19p13.2 locus contributing to the gene-based association signal in males (excel file). Acronyms: *b*: beta; s.e.: standard error.

Supplementary Table S5. Genes associated with HIV control in the full sample, males only and females only analyses.

(excel file).

Supplementary Table S6. SNPs with significant differential effect in males vs females given by variants with positive (spVL increasing) effect observed in females compared to negative (spVL decreasing) or neutral effect in males. Markers with statistically significant differential effect are shown in bold. *b*= beta, s.e.=standard error (excel file).

Supplementary Table S7. SNPs with significant differential effect on HIV control in males vs females given by variants with positive (favorable) effect observed in females compared to negative (unfavorable) or neutral effect in males. Markers with statistically significant differential effect are shown in bold. *b*= beta, s.e.=standard error (excel file).

Supplementary Table S8. Functionally mapping of genes with differential effect on spVL in males vs. females.

Supplementary Table S9. Functionally mapping of genes with differential effect on HIV control in males vs. females.

Supplementary Figures

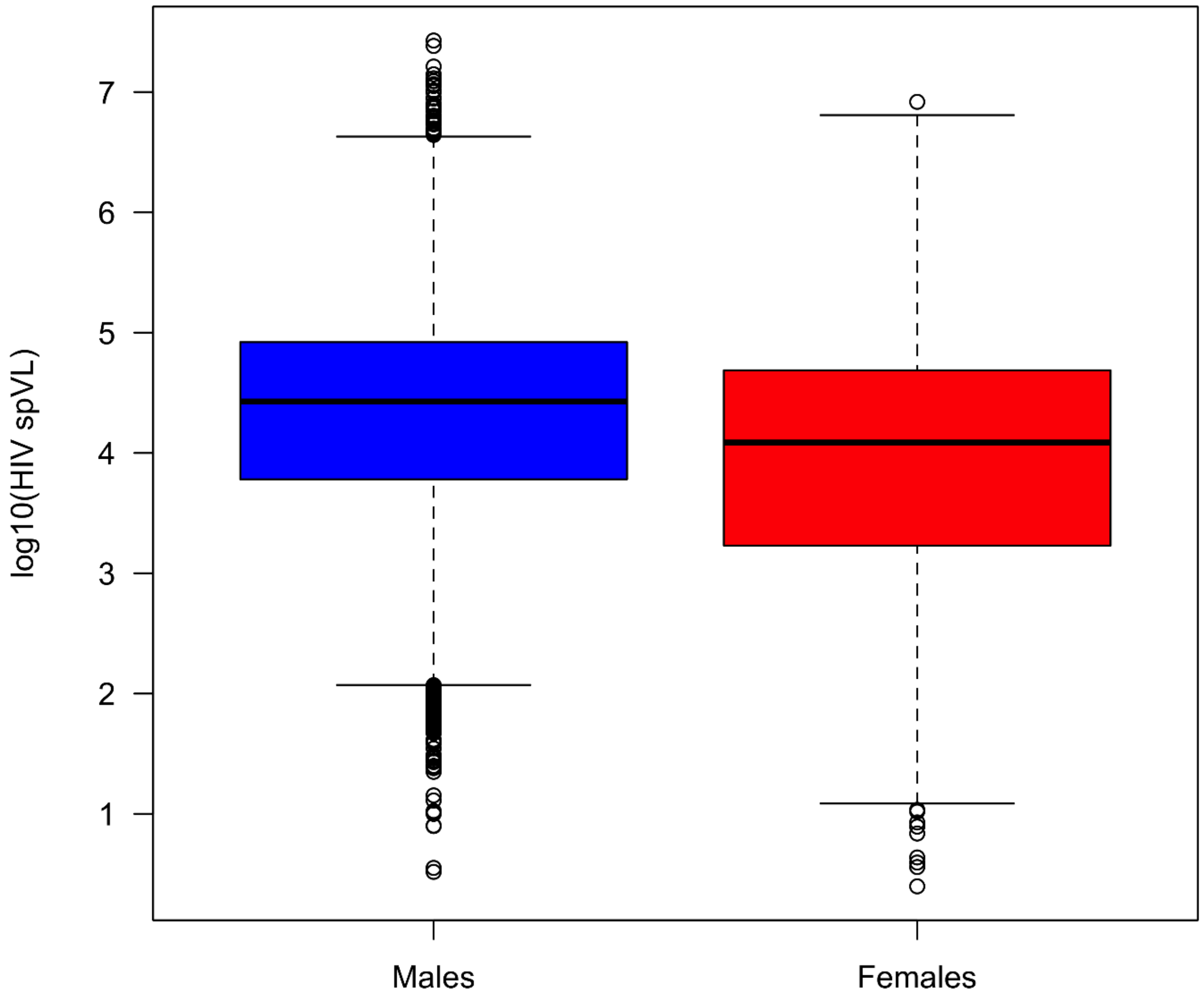

Supplementary Figure S1: Setpoint viral load differs by sex. Boxplots show the mean and quartile ranges of spVL in males (n=7,890) and females (n=1,815). Females have an ~0.36 log lower mean setpoint viral load compared to males (P value =2.4 x 10^{-41}).

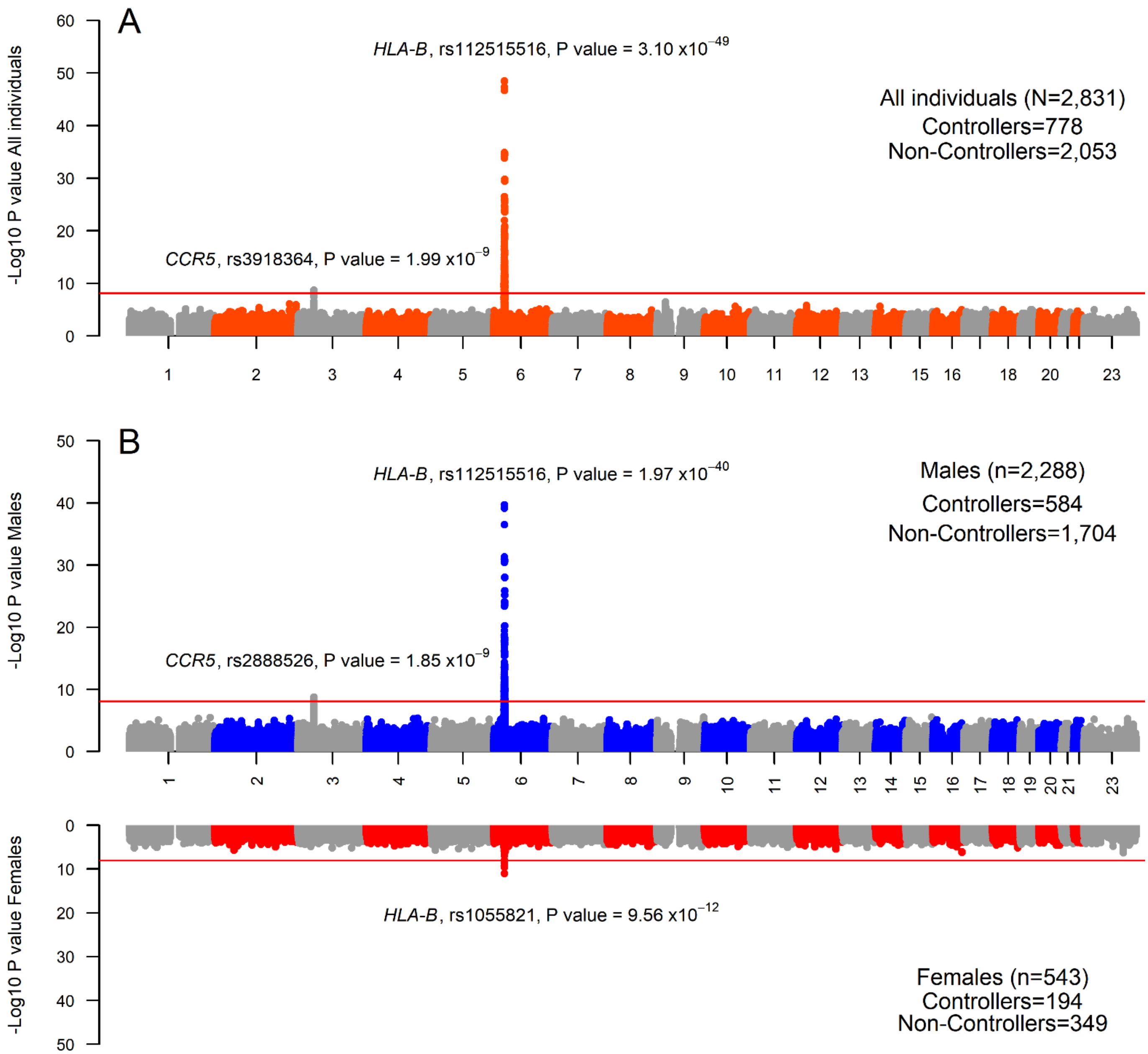

Supplementary Figure S2: Manhattan plots of genome-wide association results between common genetic variants (MAF > 0.01) and HIV control status. Association was tested using linear mixed models in the A full sample, B males only and females only. Each dot indicates a genetic variant tested for association with spVL ordered by their physical position in the genome (x-axis) and strength of association (-log10(P value), y-axis). Coordinates are based on GRCh38/hg38. The dashed line indicates statistical significance accounting for multiple comparisons (P value =8.3 x 10^{-9}). The signals on chromosome 3 and chromosome 6 correspond to the *CCR5* and *HLA* class I regions respectively.

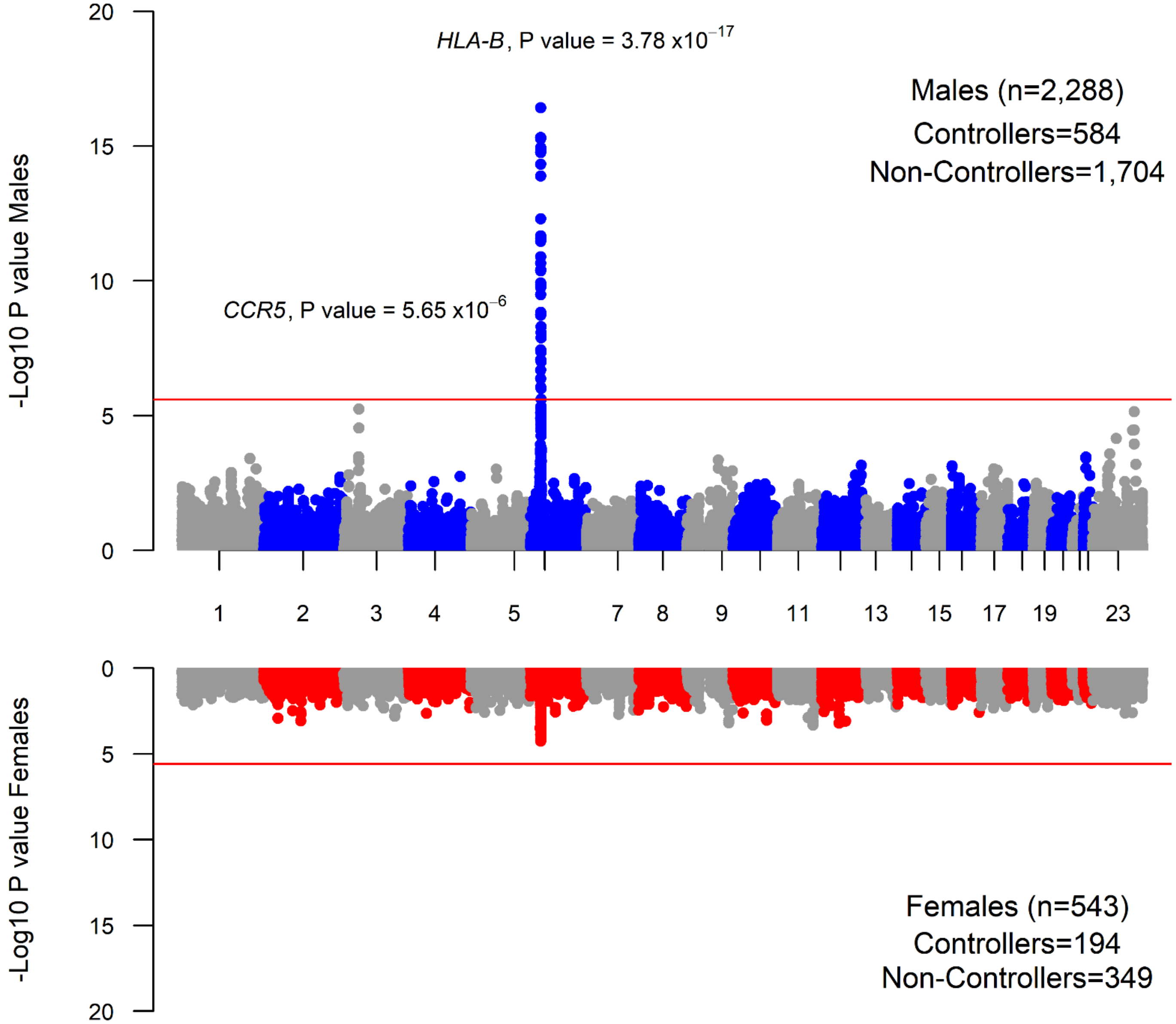

HLA-B, P value = 3.78 x10^-17
Males (n=2,288)
Controllers=584
Non-Controllers=1,704
CCR5, P value = 5.65 x10^-6
-Log10 P value Males
20
15
10
5
0
1
2
3
4
5
7
8
9
11
13
15
17
19
23
-Log10 P value Females
Females (n=543)
Controllers=194
Non-Controllers=349

Supplementary Figure S3: Manhattan plots of gene-based association with HIV control in males (upper panel) and females (lower panel). SNPs were assigned to the genes obtained from Ensembl build 85 (all genes). Each dot indicates a gene tested for association with spVL ordered by their physical position in the genome (x-axis) and strength of association (-log10(p-value), y-axis) as calculated by MAGMA (see Methods). Coordinates are based on GRCh37/hg19. The red dashed line indicates statistical significance accounting for multiple comparisons (P value =2.56 x 10^{-6}). Abbreviations*:* *CCR5*: C-C motif chemokine receptor 5, *HLA-B*: major histocompatibility complex, class I, B.

Supplementary Note: Members of the International Collaboration for the Genomics of HIV

Arman Bashirova[1,2], Susan Buchbinder[3], Mary Carrington[1,2], Andrea Cossarizza[4], Jacques Fellay[5,6,7], James J Goedert[8], David B Goldstein[9], David W Haas[10], Joshua T Herbeck[11], Eric O Johnson[12], Pontiano Kaleebu[13,14], William Kilembe[15], Gregory D Kirk[16], Neeltje A Kootstra[17], Alex H Kral[18], Olivier Lambotte[19,20], Ma Luo[21,22], Simon Mallal[23], Javier Martinez-Picado[24-27], Paul J McLaren[21,22], Laurence Meyer[28,29], José M Miro[27,30], Pravi Moodley[31], Ayesha A Motala[32], James I Mullins[33], Niels Obel[34], Fraser Pirie[32], Guido Poli[35], Matthew A Price[36,37], Andri Rauch[38], Manjinder S Sandhu[39,40], Ioannis Theodorou[41], Alexandra Trkola[42], Bruce D Walker[1,43], Cheryl A Winkler[44], Steven M Wolinsky[45], Jean-François Zagury[46].

1. Ragon Institute of MGH, MIT and Harvard, Boston, Massachusetts, USA
2. Basic Science Program, Frederick National Laboratory for Cancer Research, National Cancer Institute, Frederick, Maryland, USA and Laboratory of Integrative Cancer Immunology, Center for Cancer Research, National Cancer Institute, Bethesda, Maryland, USA
3. Bridge HIV, San Francisco Department of Public Health, San Francisco, California, USA
4. Department of Medical and Surgical Sciences for Children and Adults, University of Modena and Reggio Emilia, Modena, Italy
5. Global Health Institute, School of Life Sciences, École Polytechnique Fédérale de Lausanne, Lausanne, Switzerland
6. Swiss Institute of Bioinformatics, Lausanne, Switzerland
7. Precision Medicine Unit, Biomedical Data Science Center, Lausanne University Hospital (CHUV) and University of Lausanne, Lausanne, Switzerland

8. Epidemiology and Biostatistics Program, Division of Cancer Epidemiology and Genetics, National Cancer Institute, National Institutes of Health, Bethesda Maryland USA
9. Institute for Genomic Medicine, Columbia University, New York, New York, USA
10. Vanderbilt University School of Medicine, Nashville, Tennessee, USA
11. Department of Global Health, University of Washington, Seattle, Washington, USA
12. Center for Omics Discovery and Epidemiology, RTI International, North Carolina, USA
13. Medical Research Council/Uganda Virus Research Institute & London School of Hygiene and Tropical Medicine, Uganda Research Unit, Uganda
14. London School of Hygiene and Tropical Medicine, London, United Kingdom
15. Zambia Emory HIV Research Project, Lusaka, Zambia
16. Department of Epidemiology, Johns Hopkins University, Baltimore, Maryland, USA
17. Department of Experimental Immunology, Amsterdam UMC, University of Amsterdam, Netherlands
18. Community Health Research Division, RTI International, Berkeley, California, USA
19. Université Paris Saclay, Inserm UMR1184, CEA, Le Kremlin-Bicêtre, France
20. APHP, department of clinical immunology, Bicêtre Hospital, Le Kremlin-Bicêtre, France
21. National Microbiology Laboratory, Public Health Agency of Canada, Winnipeg, Canada
22. Department of Medical Microbiology and Infectious Diseases, University of Manitoba, Winnipeg, Canada
23. Institute for Immunology & Infectious Diseases, Murdoch University and Pathwest, Perth, Australia
24. IrsiCaixa AIDS Research Institute, Badalona, Spain
25. University of Vic - Central University of Catalonia, Vic, Spain

26. Catalan Institution for Research and Advanced Studies, Barcelona, Spain
27. CIBERINFEC, Instituto de Salud Carlos III, Madrid, Spain
28. INSERM U1018, Université Paris-Saclay 11, Le Kremlin Bicêtre, France,
29. AP-HP, Hôpital de Bicêtre, Département d'épidémiologie, Le Kremlin Bicêtre, France
30. Infectious Diseases Service. Hospital Clinic – Institut d'Investigacions Biomèdiques August Pi I Sunyer (IDIBAPS). University of Barcelona. Barcelona, Spain
31. National Health Laboratory Service, South Africa and University of KwaZulu-Natal, Durban, South Africa
32. Department of Diabetes and Endocrinology, School of Clinical Medicine, University of KwaZulu-Natal, Durban South Africa
33. Department of Microbiology, University of Washington, Seattle, Washington, USA
34. Department of Infectious Diseases, Copenhagen University Hospital, Rigshospitalet, Copenhagen, Denmark
35. Division of Immunology, Transplantation and Infectious Diseases, San Raffaele Scientific Institute, Milan, Italy and Vita-Salute San Raffaele University, School of Medicine Milan, Italy
36. International AIDS Vaccine Initiative, New York City, New York, USA
37. Department of Epidemiology and Biostatistics, University of California, San Francisco, California, USA
38. Department of Infectious Diseases, Inselspital, University Hospital Bern, Bern, Switzerland
39. Department of Epidemiology & Biostatistics, School of Public Health, Imperial College London, UK
40. Omnigen Biodata, Cambridge, UK

41. Laboratoire d'Immunologie et Histocompatibilité, Pôle Biologie-Pathologie-Physiologie, Hôpital Saint-Louis, Paris, France
42. Institute of Medical Virology, University of Zurich, Zurich, Switzerland
43. Howard Hughes Medical Institute, Chevy Chase, Maryland, USA
44. Basic Research Laboratory, Molecular Genetic Epidemiology Section, Center for Cancer Research, NCI, Leidos Biomedical Research, Inc., Frederick National Laboratory, Frederick, Maryland, USA
45. Division of Infectious Diseases, The Feinberg School of Medicine, Northwestern University, Chicago, Illinois, USA
46. Laboratoire Génomique, Bioinformatique, et Applications, Chaire de bioinformatique, Conservatoire national des arts et métiers, Paris, France
